# Model-free optical processors using in situ reinforcement learning with proximal policy optimization

**DOI:** 10.1038/s41377-025-02148-7

**Published:** 2026-01-01

**Authors:** Yuhang Li, Shiqi Chen, Tingyu Gong, Aydogan Ozcan

**Affiliations:** 1 Electrical and Computer Engineering Department, University of California, Los Angeles, CA USA; 2 California NanoSystems Institute (CNSI), University of California, Los Angeles, CA USA; 3 Computer Science Department, University of California, Los Angeles, CA USA; 4 Bioengineering Department, University of California, Los Angeles, CA USA

**Keywords:** Imaging and sensing, Adaptive optics

## Abstract

Optical computing holds promise for high-speed, energy-efficient information processing, with diffractive optical networks emerging as a flexible platform for implementing task-specific transformations. A challenge, however, is the effective optimization and alignment of the diffractive layers, which is hindered by the difficulty of accurately modeling physical systems with their inherent hardware imperfections, noise, and misalignments. While existing in situ optimization methods offer the advantage of direct training on the physical system without explicit system modeling, they are often limited by slow convergence and unstable performance due to inefficient use of limited measurement data. Here, we introduce a model-free reinforcement learning approach utilizing Proximal Policy Optimization (PPO) for the in situ training of diffractive optical processors. PPO efficiently reuses in situ measurement data and constrains policy updates to ensure more stable and faster convergence. We validated our method through both simulations and experiments across a range of in situ learning tasks, including targeted energy focusing through a random diffuser, image generation, aberration correction, and optical image classification, demonstrating in each task better convergence and performance. Our strategy operates directly on the physical system and naturally accounts for unknown real-world imperfections, eliminating the need for prior system knowledge or modeling. By enabling faster and more accurate training under realistic experimental constraints, this in situ reinforcement learning approach could offer a scalable framework for various optical and physical systems governed by complex, feedback-driven dynamics.

## Introduction

As the demand for faster, more efficient artificial intelligence (AI) computation grows, physical neural networks (PNNs), which perform computation using analog systems, have emerged as an alternative to traditional digital processors. By leveraging physical processes such as light propagation, electrical response, or acoustic vibration, PNNs offer the potential for ultra-low-latency, energy-efficient inference and edge computing^1–4^. This paradigm is especially promising in the domain of optical computing, where the input information is rapidly processed using thin optical components^5^. A variety of optical platforms have been explored, including diffractive optical networks^6–9^, integrated photonic neural networks^10–13^, and optical reservoir computing^14–17^, each offering unique advantages for accelerating light-based computation.

**Fig. 1:**
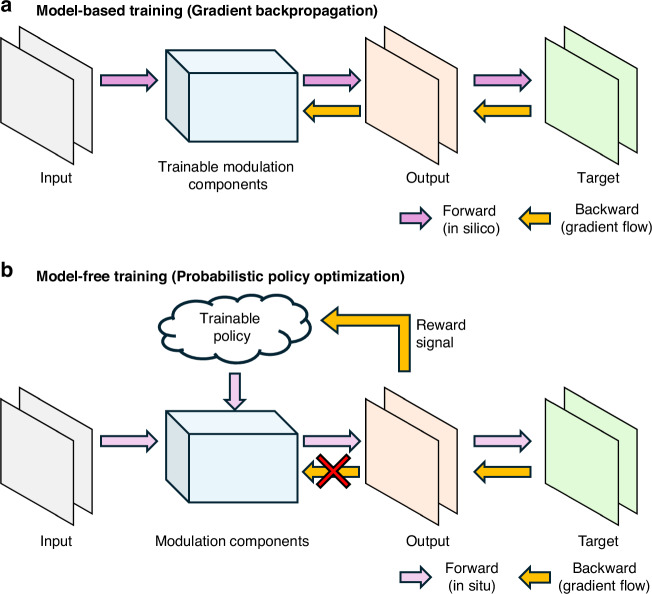
Conceptual overview of model-based and model-free optical learning. **a** Model-based training relies on gradient backpropagation through a differentiable in silico model of the system. Error gradients are calculated and propagated backward to directly update the system’s trainable components. **b** Model-free training employs probabilistic policy optimization to work directly with the physical hardware (in situ). A trainable policy generates system parameters, and a reward signal calculated from the measured output is used to update the policy. This reinforcement learning-based method treats the physical system as a black box and does not require a differentiable model or gradient flow through the components

Designing optical computing systems typically involves a two-step process: first, digitally emulating and optimizing the physical parameters of the hardware in a simulated environment; and second, deploying the optimized configuration into a real-world physical system^6,13,18–20^. In silico training methods rely on physics-based forward models or neural network surrogates to construct digital twins of PNNs, which are then optimized in silico for specific tasks, as shown in Fig. 1a. However, this simulation-driven in silico approach faces inherent limitations. Accurately modeling the physical system is often undermined by the simulation-to-reality gap^21–23^; real-world systems are affected by noise, optical misalignments, and fabrication or device imperfections that are difficult to accurately model or know a priori^24^. Even when a reasonable model exists, simulating physical processes requires fine discretization of space and time, making optimization computationally expensive and susceptible to numerical errors^25,26^. Some of the recent approaches have explored a hybrid strategy by combining digital and physical elements during training. In these physics-aware training methods, a fixed physical system performs the forward pass, while a co-optimized digital model computes the backward pass via gradient-based updates^1,27^. Although hybrid strategies that combine physical feedback with a digital model relax the strict requirement of having a perfect digital twin, they still depend on maintaining a close correspondence between the digital and physical worlds, a challenge that grows with system complexity. If the system drifts or exhibits unmodeled/unknown dynamics or aberrations, the gradient estimates become unreliable.

To eliminate this dependence on accurate models, model-free training algorithms have recently gained attention^2,28–33^. In particular, in situ training methods—where the optimization is performed directly on the physical hardware—have begun to emerge. One set of approaches involves optical implementations of backpropagation, demonstrated in both linear and nonlinear optical networks, where gradients are computed using forward and backward light propagation. While powerful, these approaches demand precise optical alignments, which might limit their practical applications in diverse or noisy experimental settings^29,34^. More flexible model-free optimization techniques, such as gradient-free and evolutionary algorithms, have also been explored^35–38^. These include methods based on perturbation strategies—such as Simultaneous Perturbation Stochastic Approximation (SPSA)—and those based on sampling from distributions^32,37,39–41^, including Genetic Algorithms (GA), Evolutionary Strategies (ES), and swarm optimization. Reinforcement learning (RL) has also been applied to the optical domain, including integrated photonics and optical communication networks^42–45^. More recently, score-function-based gradient estimators have also been used to train optical systems, where policy gradient (PG) methods, including natural gradient variants that refine the update direction, directly optimize the system’s response without an explicit physical model^28,46–50^; for example, a fully autonomous and high-performance optical neural network^51^ was demonstrated using a vertical-cavity surface emitting laser for model-free training of optical processors suitable for various inference tasks. These approaches interact directly with the physical system for the forward pass and use reward signals to guide updates—without requiring explicit gradient computation through the hardware; see Fig. 1b. Although these efforts have shown promising results, a key bottleneck shared by all these in situ model-free approaches is the relatively high cost of physical measurements. Unlike digital computations, capturing each physical measurement is a slow, sequential process constrained by the speed of optical hardware, e.g., spatial light modulators (SLMs) and optical sensors. This makes data collection time-consuming. Moreover, standard methods typically discard collected samples after each update, resulting in inefficient data usage and unreliable gradient estimates. As a result, updating the policy parameters based on each new batch of measurements can make data inefficiency a critical issue. Furthermore, standard PG methods often suffer from unstable convergence, significantly compounding this data inefficiency problem by requiring more iterations to reach an optimal solution. These drawbacks are especially problematic in physical systems, where each policy update requires real-world measurements and is therefore time-consuming and resource-intensive.

Here, we introduce a model-free, in situ reinforcement learning framework based on Proximal Policy Optimization (PPO) for training optical processors. PPO improves optical training efficiency by enabling multiple digital updates per batch of captured optical measurements and ensures stable convergence by constraining policy changes, even under noisy experimental conditions^52^. We numerically and experimentally demonstrated the effectiveness of this in situ optical learning approach across a variety of tasks—including energy focusing through a random, unknown diffuser, holographic image generation, aberration correction, and optical image classification. Crucially, our PPO-based method achieved substantially faster convergence and improved final performance across all tasks, without relying on physical system models or explicit gradient calculations. We anticipate that this robust, data-efficient training strategy will significantly advance the practical deployment of optical processors and it offers a generalizable framework for optimizing complex, feedback-driven physical systems.

## Results

**Fig. 2:**
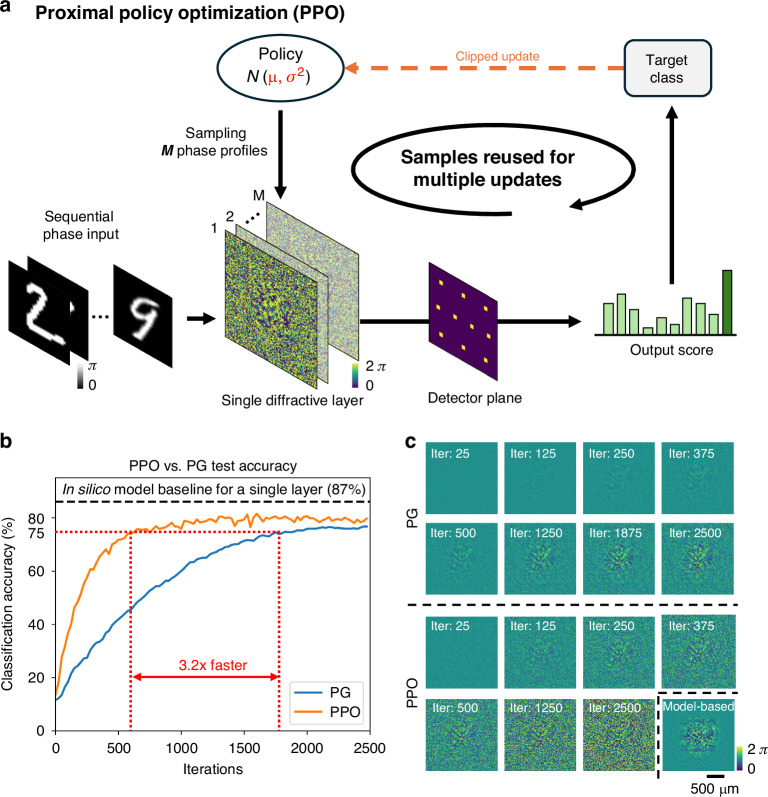
Proximal Policy Optimization (PPO) for in situ training of a diffractive optical classifier. **a** Schematic of the PPO training framework. A policy generates phase patterns for a single diffractive layer. The physical output is measured, and PPO uses a clipped update rule and reuses the measured data for multiple optimization steps to efficiently train the policy. **b** Numerical test accuracy comparison between PPO and standard Policy Gradient (PG). PPO achieves higher classification accuracy and converges faster. The dashed line denotes the performance thresholds, defined as 85% of the in silico model performance for a single-layer diffractive configuration. **c** Evolution of the learned diffractive phase patterns at different iterations of PPO and PG compared with the in silico model (denoted as Model-based)

Policy gradient is a class of reinforcement learning algorithms that directly optimize a parameterized policy, i.e., a function that maps system states to actions, by adjusting its parameters to maximize an expected reward. These methods estimate gradients that guide the policy to iteratively increase the likelihood of actions yielding higher rewards, while decreasing the likelihood of actions with lower rewards. In our in situ optical learning framework, we utilized PPO to achieve stability, data efficiency, and enhanced performance across various optical tasks. As shown in Fig. 2a, the PPO-based training strategy improves the in situ reinforcement learning process by enabling the reuse of output measurements. In each training round, we sample $M$ phase profiles ${\left\{{\varphi }_{j}\right\}}_{j=1}^{M}$ from the current policy ${\pi }_{\theta }$, display them sequentially on the SLM, and record the resulting optical measurements, corresponding to a certain desired optical task. These measurements are then used to estimate the advantage function and compute the PPO loss^53^; see “Methods” for details. PPO performs multiple optimization steps using the same set of measured physical/experimental data. This significantly improves data efficiency—an essential benefit in time-constrained in situ optical experiments. To prevent these repeated updates from causing instability or divergence, we used a clipped surrogate objective that constrains how much the policy can change in each step. The PPO objective is defined as^52^:

1 $${J}^{{PPO}}\left(\theta \right)=-{{\mathbb{E}}}_{\varphi \sim {\pi }_{\theta }}\left[{\text{min}}\left(r\left(\varphi ;\theta \right){A}^{{\prime} }\left(\varphi \right),{\text{clip}}\left(r\left(\varphi ;\theta \right),1-\epsilon ,1+\epsilon \right){A}^{{\prime} }\left(\varphi \right)\right)\right]$$

where $r\left(\varphi ;\theta \right)=\frac{{\pi }_{\theta }\left(\varphi \right)}{{\pi }_{{\theta }_{{old}}}\left(\varphi \right)}$ is the probability ratio between the current policy ${\pi }_{\theta }\left(\varphi \right)$ and previous policy ${\pi }_{{\theta }_{{old}}}\left(\varphi \right)$, and ${A}^{{\prime} }\left(\varphi \right)$ denotes the normalized advantage function, which measures how much better or worse a sampled phase profile $\varphi$ performed compared to the average (see “Methods” for details). The ${\rm{clip}}$ operator constrains the probability ratio of $r(\varphi ;\theta )$ to the range $[1-\epsilon ,1+\epsilon ]$, where $\epsilon$ is a hyperparameter limiting the extent of the policy update to ensure stability. The core clipping mechanism prevents overly large updates by enforcing a conservative change in the policy distribution, thereby ensuring stable convergence even when using repeated samples of physical measurement data. We performed this surrogate loss optimization for $K$ iterations using the same batch of collected data before sampling new data. By reusing collected data and preventing overly large policy shifts, PPO offers a practical balance between exploration and exploitation, making it well-suited for data-constrained experimental scenarios.

We first validated our PPO-based model-free reinforcement learning strategy in a simulated optical classification task, where a diffractive layer was optimized to classify phase-encoded MNIST digits^54^ (see “Methods” for details). Figure 2b quantitatively compares the convergence of PG and PPO. The PPO strategy using a single diffractive layer achieved a final test accuracy of ~80% and 3.2 times faster than PG. This accelerated convergence demonstrates PPO’s effectiveness in optimizing complex optical transformations through reinforcement learning. Furthermore, Fig. 2c visualizes the evolution of the learned phase patterns over training iterations, showing that PPO rapidly learned clear and structured phase patterns. This comparative analysis confirms PPO’s superiority in both convergence speed and training stability, establishing it as a compelling reinforcement learning approach for optical processor design and implementation.

**Fig. 3:**
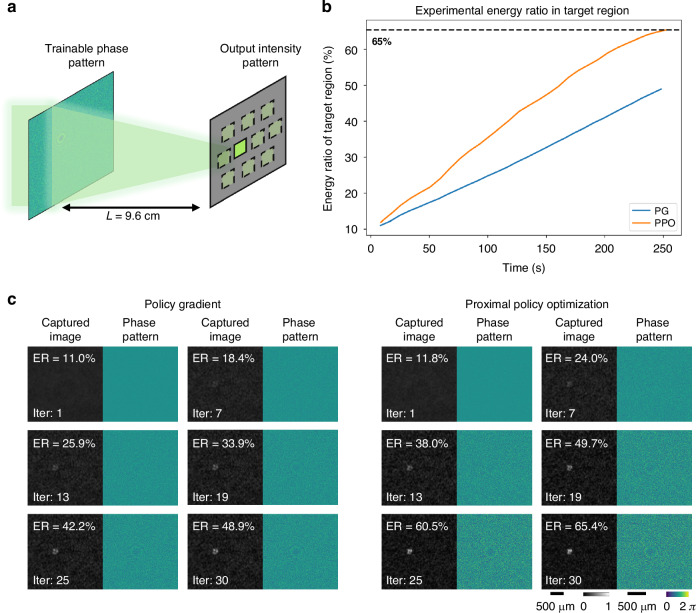
Experimental reinforcement learning results for in situ optimization of targeted energy focusing. **a** A schematic of the physical setup, wherein a trainable phase pattern is optimized in real-time to focus light onto a designated target area while minimizing energy in the other 9 selected areas. **b** The energy ratio (ER) concentrated in the target/desired region is plotted as a function of the experimental reinforcement learning time. **c** Time-lapse visualization of the captured intensity patterns and the corresponding SLM phase patterns for PG (left) and PPO (right) during the optimization

**Fig. 4:**
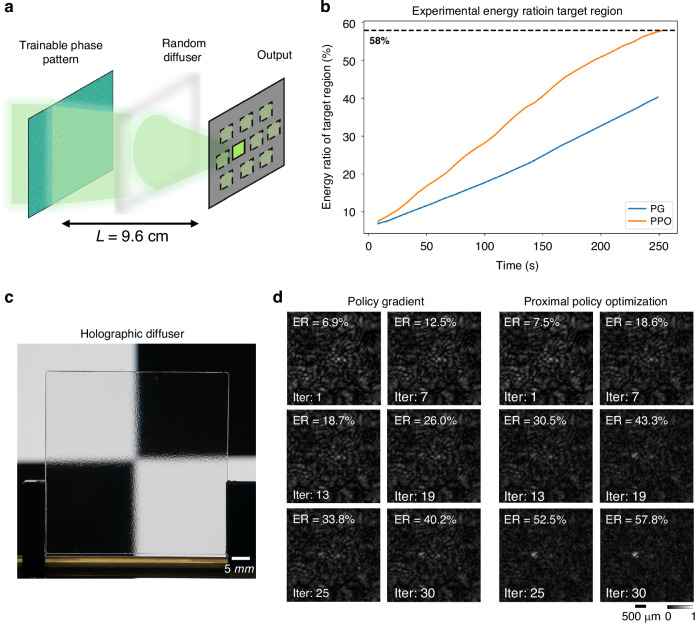
Experimental reinforcement learning results for in situ optimization of targeted energy focusing with a random, unknown diffuser inserted between the SLM and the image sensor plane. **a** A schematic of the physical setup, wherein a trainable phase pattern is optimized in real-time to focus light through an unknown, random diffuser onto a designated target area while minimizing energy in the other 9 selected areas. **b** The energy ratio (ER) concentrated in the target region is plotted as a function of experimental reinforcement learning time. **c** Photograph of the unknown, random diffuser used in the experiment. **d** Time-lapse evolution of the captured intensity patterns for PG (left) and PPO (right) during the optimization with the random diffuser inserted

Following these simulations, we experimentally evaluated the performance of our PPO-based in situ reinforcement learning framework on an energy focusing task as shown in Fig. 3a. A trainable phase pattern displayed on an SLM modulated the incoming wavefront, and the resulting intensity distribution was recorded on an image sensor plane subdivided into ten detection regions. The objective was to maximize the energy in the designated target region relative to the total energy across all ten regions. This selective beam focusing task provides a benchmark for precisely shaping and controlling light using diffractive optics. Figure 3b demonstrates that PPO achieved significantly faster and more effective energy focusing onto target/desired regions. This advantage is visually confirmed in Fig. 3c, where the PPO-trained system produced a high-intensity focal spot earlier and with greater clarity. We further conducted experiments with a random, unknown diffuser (Fig. 4c) inserted between the SLM and the image sensor plane, as illustrated in Fig. 4a. The curves in Fig. 4b and the results in Fig. 4d further highlight PPO’s robustness, demonstrating its ability to maintain effective focusing even in the presence of unknown optical perturbations introduced by a random diffuser. In addition, our simulation-based analysis (Supplementary Fig. S1a) revealed that the learned phase pattern, although trained with a specific diffuser at a fixed position, maintained an energy focusing ratio of >50% within a diffuser lateral shift of approximately $\pm 16$ µm. These results provide evidence that our model-free PPO framework can enable robust and highly efficient in situ optimization of a diffractive optical processor. We further fine-tuned the trained phase pattern under different diffuser displacements for a limited number of learning iterations, as shown in Supplementary Figs. S1b-d. Only 10 iterations were sufficient to achieve an energy ratio above 50% for displacements within $\pm 40$ µm. Furthermore, with 35 iterations, the energy ratio was maintained above 50% across the entire tested lateral misalignment range of $\pm 160$ *µm*. We also numerically compared our method with respect to PG and genetic algorithms (GA). As shown in Supplementary Fig. S2a, PPO achieved faster and more stable convergence, whereas GA exhibited only transient early improvements and failed to reach comparable final performance. We further compared PPO with PG and GA on another task, as shown in Supplementary Fig. S2b, where phase aberrations were introduced at the encoded phase pattern. PPO once again showed faster convergence and better performance within a limited number of optimization iterations. These results and analyses further confirm the advantages of PPO for efficient and robust in situ training of diffractive optical processors.

**Fig. 5:**
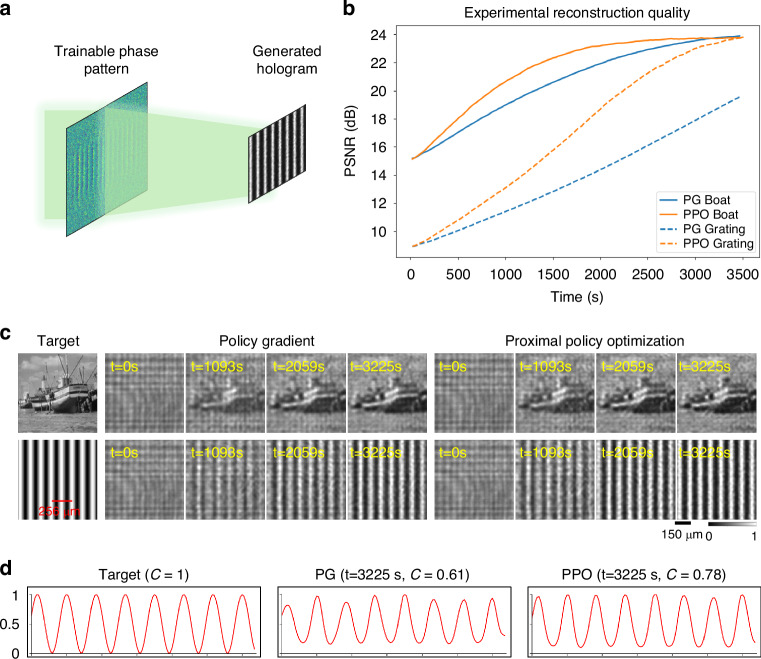
Experimental reinforcement learning results for in situ holographic image generation. **a** Schematic of the experimental setup for holographic image projection, where a trainable phase pattern generates a selected target image (the initial condition at t = 0 s is a uniform phase pattern). **b** Reconstruction quality (PSNR) over time for two target images, a ‘Boat’ and a ‘Grating’. PPO (shown with orange lines) consistently achieves higher fidelity faster than PG (blue lines). **c** Visual comparison of holographic image quality at different time points. PPO reconstructions (right) are clearer and more accurate than PG reconstructions (middle) at all stages. **d** A cross-sectional intensity profiles of the ‘Grating’ pattern. Contrast values (C) were calculated, showing that PPO achieves a higher contrast

We next evaluated our in situ reinforcement learning framework on a holographic image generation task, where a phase-only SLM was optimized to produce a target image at the sensor plane, as shown in Fig. 5a. We experimentally tested the system on two distinct targets: a synthetic grating and a natural image (“Boat”). As shown in Fig. 5b, PPO achieved a higher Peak Signal-to-Noise Ratio (PSNR) in less training time. The visual evolution of the grating image generation in Fig. 5c further confirms that PPO produced sharper, higher-fidelity images using fewer iterations. This ability to efficiently learn complex optical transformations in situ highlights our method’s potential for applications such as holographic displays and lensless imaging.

**Fig. 6:**
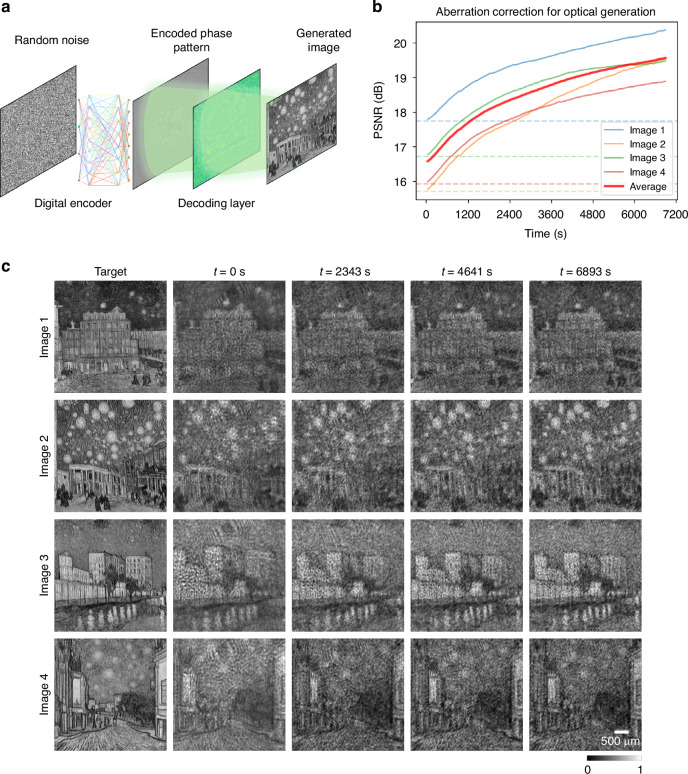
In situ correction of misalignment-induced aberrations for synthesizing novel images through an optical generative model^9^. **a** Schematic of the optical generative model, where a digital encoder transforms random noise into an encoded phase pattern, which is displayed on an SLM. This encoded phase pattern, projected by an SLM, then passes through a separate decoding SLM to form a novel image; different than Fig. 5, here the target images are novel, synthesized by an optical generative model. **b** Reconstruction quality (PSNR) as a function of the in situ training time for four different novel images created from random noise. The average performance (red line) shows a consistent improvement, demonstrating successful in situ aberration correction through reinforcement learning. **c** Visual results of the RL-based optimization process. The generated novel images from random input noise, initially aberrated at *t* = 0 s, become progressively clearer and more recognizable over time as the system learns in situ to compensate for aberrations

We further extended the PPO-based reinforcement learning framework to an in situ image improvement task designed to correct for system aberrations and random misalignments. For these experiments, we used a randomly misaligned optical generative model—comprising a digital encoder and an optical decoder (SLM)—that were jointly pre-trained in silico^9^. This optical generative model used a digital encoder with 85 M parameters, and the learning of the student optical model was distilled from a fine-tuned diffusion model trained on 800 Van Gogh paintings^55^—which was used as the teacher model (only during training). The student optical model, trained with the teacher, provides an optical phase-only generative seed of 1000×1000 pixels for each new image to be generated in a snapshot, calculated from random noise of size 80×80. The snapshot student model’s optical decoder (size 800 × 800) was jointly trained with the digital encoder. In other words, different from Fig. 5, here the image to be projected is a novel image created by a snapshot optical generative model. After the training of the snapshot optical generative model, to mitigate the random misalignment-related aberrations of the optical hardware and improve the output quality of the generated images, we used PPO to fine-tune and adaptively optimize the phase pattern of the optical decoder SLM, directly on the hardware (Fig. 6a). This in situ reinforcement learning process allows the generative system to learn and compensate for the physical imperfections/misalignments in the optical hardware. Our experimental results demonstrate a consistent improvement in image quality across various image generation tasks. As quantified in Fig. 6b, this PPO-based calibration led to a marked increase in PSNR. The visual results of these experiments, shown in Fig. 6c, also illustrate this enhancement. With the in situ reinforcement learning, an improvement in image clarity and a more accurate match to the corresponding in silico-generated images can be observed. These experimental results further establish our PPO-based reinforcement learning framework as a powerful and efficient method for correcting experimental aberrations and misalignments in dynamic task-specific optical systems.

**Fig. 7:**
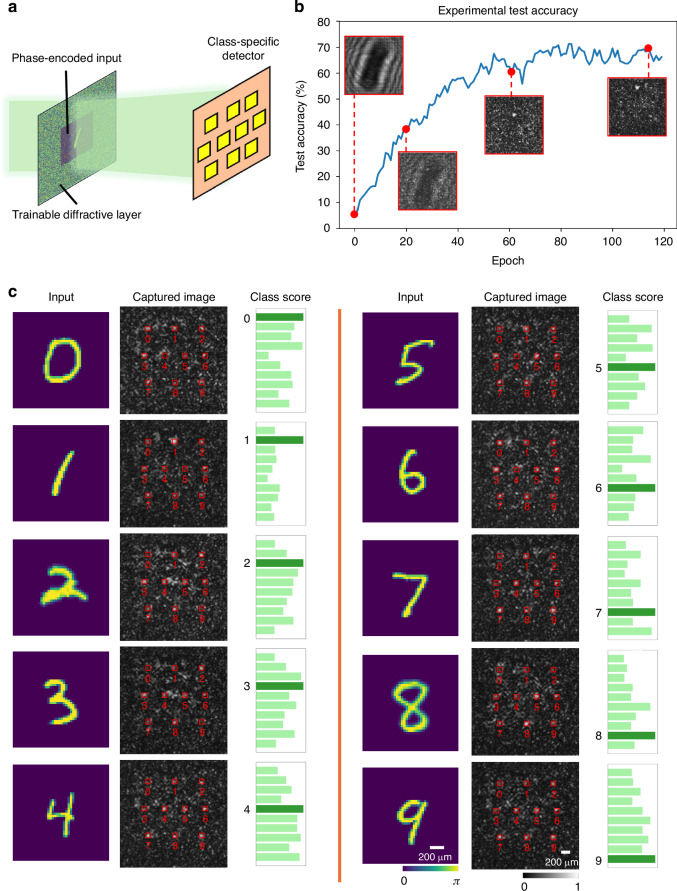
Experimental demonstration of in situ reinforcement learning for an all-optical diffractive image classifier. **a** A schematic of the optical setup where a trainable diffractive layer processes phase-encoded input images (to be classified and never seen before) and directs the input light to a grid of 10 class-specific detectors used for all-optical image classification. **b** Experimental test accuracy plotted against the in situ training epoch number, showing the learning progression of the physical system. Insets visualize the evolution of the output intensity patterns at various stages of the RL process; once converged, the learned phase pattern is fixed and can be used to classify new test objects never seen before. **c** Classification test examples for ten different handwritten digits {0, 1, …, 9}, never seen before. The final learned phase profile successfully directs the light from each input digit to its correct corresponding detector, as confirmed by the captured images and class scores

Finally, we validated the general applicability of our PPO-based in situ learning framework for optical computing on an image classification task, specifically classifying handwritten digits (MNIST dataset). As a proof of concept, the experimental setup (Fig. 7a) used an 800 × 800 region of the SLM as a single layer, where the input digit was simultaneously phase-encoded directly onto the same SLM plane. The trainable diffractive layer was optimized in situ to learn the class-specific patterns and smartly guide the optical energy toward the detector corresponding to the correct digit class, i.e., performing all-optical image classification. Figure 7b plots the experimental test accuracy over in situ training epochs. We observed rapid improvement in classification performance during the initial training stages of the reinforcement learning process. Insets show examples of the captured intensity patterns at selected epochs, clearly demonstrating how initially indistinct outputs evolved into clear and discriminative patterns as the in situ training progresses. Ten illustrative classification examples for handwritten digits from “0” to “9” are visualized in Fig. 7c. Each digit, encoded as a phase input image, was transformed through the learned phase profile. The experimentally captured images reveal clear peaks at the correct class-specific detector positions. The corresponding class scores, derived from these measured intensities, confirm that each digit was correctly identified by the diffractive optical processor. Additional simulation results comparing PPO, PG, and GA are also provided in Supplementary Fig. S2c, where PG exhibited slower convergence and GA failed to train successfully. These results underscore the effectiveness of our PPO-based in situ learning method for directly training optical processors, offering a robust pathway for developing physical neural networks without reliance on digital twins or model-based error back-propagation.

## Discussion

In this work, we introduced a practical and efficient framework for the in situ training of diffractive optical processors using proximal policy optimization. Our model-free PPO-based reinforcement learning method effectively bypasses the gap between simulations and real-world experiments by learning directly from physical measurements. One of the key strengths of our framework is its significant improvement in in situ learning speed: by enabling multiple updates from a single batch of experimental data, PPO significantly reduces the number of physical measurements needed for convergence. The clipped surrogate objective used in PPO plays a pivotal role in this acceleration. By preventing large policy updates and enforcing stable improvement, PPO-based reinforcement learning ensures steady and robust convergence even under noisy, limited, or imperfect measurement conditions. This robustness allows for more aggressive optimization without sacrificing stability, further contributing to the speed-up. We validated this improvement through both simulations and experiments. For example, in our optical image classification task, simulations showed that PPO achieved more than $3\times$ faster convergence compared to other RL methods. Similar advantages in learning speed were also observed in experimental results of targeted energy focusing and holographic image generation. Moreover, we also demonstrated the real-world experimental applicability of PPO-based reinforcement learning for in situ aberration corrections and optical image classification tasks, where the PPO-based training enabled robust and efficient system optimization directly from physical feedback.

Furthermore, we examined the sensitivity of our PPO-based framework to hyperparameters such as learning rate, sampling size, and the clipping factor $\epsilon$, as shown in Supplementary Fig. S3. Across a broad range of settings, the training consistently converged with only minor differences in speed, indicating that the method was generally robust and did not require extensive hyperparameter tuning. It is also worth mentioning that our in situ framework inherently compensates for some of the optical variations, such as wavelength shifts or defocus, if they are present during training, since the optimization is performed directly on the hardware, and it indirectly integrates all these factors or variations into the learned phase patterns. Notably, for all the results and analyses reported above, the wavelength was not explicitly specified or used in the training algorithm, as it is model-free. We further tested the robustness of the trained system using numerical simulations, when such variations occurred “after” training. As shown in Supplementary Figs. S4a,b, an optical classifier trained at $532$ nm maintained > 78.9% classification accuracy across a wavelength range of $520-545$ nm, and preserved >60% accuracy under detector-plane axial defocus of approximately ±0.5 *cm*. While performance may degrade for larger amounts of shifts, our framework allows rapid in situ retraining or fine-tuning to efficiently adapt to new operating conditions, ensuring continued robust performance in a model-free environment.

We also investigated the all-optical image classifier’s robustness to additional phase noise, ${\phi }_{n}(x,y,t)$, representing pixel-wise phase shifts that vary across both spatial coordinates and time ($t$), modeling various sources of experimental noise. As shown in Supplementary Fig. S4c, the trained optical classifiers achieved similar performance metrics under a noise-free condition (${\phi }_{n}\left(x,y\right)=0$), a fixed phase offset (${\phi }_{n}\left(x,y\right) \sim U[\mathrm{0,2}\pi ]$), and dynamic, time-varying random phase noise with varying magnitudes (${\phi }_{n}\left(x,y,t\right) \sim U[\mathrm{0,0.2}\pi ],U[\mathrm{0,0.4}\pi ],U[\mathrm{0,0.6}\pi ]$). Although the performance degraded slightly at larger dynamic perturbation ranges, the system still maintained ~70% accuracy. Overall, these results demonstrate that our model-free reinforcement learning framework effectively adapts to systematic deviations while maintaining a stable performance despite random perturbations and noise.

However, several limitations remain for further improvements. The current policy used Gaussian distributions to sample trainable parameters of the optical system, which did not incorporate prior knowledge about the spatial properties of the physically realizable optical fields or structures. This can lead to inefficient exploration in a high-dimensional space^56^. To address this, future work could explore more sophisticated or expressive policy parameterizations, such as spatially correlated distributions or generative physical priors trained offline to guide the RL-based exploration in a physically conditioned subspace^57–59^. These approaches could substantially reduce the effective dimensionality of the search space, enabling faster learning with fewer iterations. Another promising future direction involves hybrid modeling methods that integrate coarse physical models with our data-driven PPO framework. In this case, an approximate model could guide the early stages of the training process or generate synthetic rollouts that accelerate policy learning. As PPO continues to refine the policy using real-world measurements, the physical model itself could be iteratively updated to better align with the true system dynamics^1,27,60,61^. This closed-loop refinement can allow for improved initialization, reduced data requirements, and a synergistic blend of model-based and model-free learning. Such hybrid strategies are particularly valuable in complex or partially known/characterized physical systems.

From a broader perspective, while our demonstrations have been for optical computing or information processing tasks such as image generation, aberration correction, and optical image classification, the underlying methodology is general and broadly applicable to other experimental learning tasks. Any physical system characterized by a measurable output and adjustable parameters within a feedback loop could potentially benefit from PPO-based in situ reinforcement learning. This approach is particularly promising in fields like adaptive optics, nonlinear photonics, multi-mode fiber-optics and other domains where model-free, feedback-driven optimization methods are critical for achieving robust performance in real-world environments^5,62^ that are particularly hard to model. Ultimately, this approach moves us toward intelligent, reconfigurable physical systems that can autonomously learn and adapt directly from real-time interactions with their environment.

## Materials and methods

### Optimization objective

Our diffractive optical system used an SLM to serve as both the input interface and the trainable optical modulation processor. Let $\varphi \in {{\mathbb{R}}}^{H\times W}$ denote the phase modulation pattern applied to the SLM. For a given optical input ${x}_{i}$, the system performs a transformation $f({x}_{i};\varphi )$, which corresponds to the light propagation and modulation governed by the phase mask $\varphi$. The resulting intensity distribution is measured by a digital camera. The goal is to train the system to approximate a desired optical transformation by minimizing the following loss:

2 $$L\left(\varphi \right)=\frac{1}{N}\mathop{\sum }\limits_{i=1}^{N}{\left|\left|f\left({x}_{i};\varphi \right)-{y}_{i}\right|\right|}^{2}$$

This loss is computed directly from physically measured intensities at the sensor plane. Since $f(\cdot )$ represents the actual light propagation and detection process within our experimental setup, the optimization inherently captures all system nonidealities, imperfections, aberrations, misalignments and noise factors.

### Proximal policy optimization for in situ reinforcement learning

We adopted a reinforcement learning approach by modeling the phase pattern $\varphi$ as a random variable drawn from a distribution ${\pi }_{\theta }$, where $\theta$ denotes the trainable parameters of the policy. Specifically, we chose a Gaussian distribution ${\mathscr{N}}{\mathscr{(}}\mu ,{\sigma }^{2})$ as our policy ${\pi }_{\theta }$, where $\theta$ denotes the mean ($\mu$) and the standard deviation ($\sigma$) of this distribution. The phase pattern displayed on the SLM is drawn from this Gaussian distribution. In our implementation, the mean ($\mu$) serves as the primary trainable parameter, while the standard deviation ($\sigma$) was fixed at 0.04. This standard deviation $\sigma$ controls the level of randomness (i.e., exploration) in the sampled patterns, allowing the algorithm to explore the parameter space. In this setting, the expected loss becomes:

3 $$J\left(\theta \right)={{\mathbb{E}}}_{\varphi \sim {\pi }_{\theta }}\left[L\left(\varphi \right)\right]$$

The policy gradient is estimated using the likelihood-ratio trick:

4 $${\nabla }_{\theta }{J}^{{PG}}=-{{\mathbb{E}}}_{\varphi \sim {\pi }_{\theta }}\left[A\left(\varphi \right){\nabla }_{\theta }\log {\pi }_{\theta }\left(\varphi \right)\right]$$

where the advantage function $A(\varphi )$ is defined as the negative loss:

5 $$A\left(\varphi \right)=-L\left(\varphi \right)$$

However, raw advantage estimates can vary significantly in scale across training batches or even within a batch, leading to unstable or inefficient updates. Normalizing the advantages to have a zero mean and unit variance helps mitigate this issue. Therefore, for convergence stability, the advantages are normalized across each batch to have zero mean and unit variance:

6 $${A}^{{\prime} }\left(\varphi \right)=\frac{A\left(\varphi \right)-{\mu }_{A}}{{\sigma }_{A}}$$

where ${\mu }_{A}$ is the mean of all $A\left(\varphi \right)$ values within one round of measurements, and ${\sigma }_{A}$ is the corresponding standard deviation.

In our implementation, the data acquisition speed was limited to ~120 ms per image, primarily due to the SLM response time and the need for longer exposure to ensure a good signal-to-noise ratio under limited laser power. In contrast, updating the policy parameters digitally required only a few milliseconds, making it negligible compared to the image acquisition time. By minimizing a surrogate objective with a clipped probability ratio defined in Eq. 1, PPO achieved fast convergence and improved performance.

Our RL-based in situ training loop consists of the following steps:

1. *Sampling*: Sample $M$ phase masks ${\left\{{\varphi }_{j}\right\}}_{j=1}^{M} \sim {\pi }_{{\theta }_{{old}}}$
2. *Physical Measurement*: For each ${\varphi }_{j}$, perform physical measurements over a batch of $B$ inputs ${\left\{{x}_{i}\right\}}_{i=1}^{B}$, yielding $N=M\times B$ data points in total.
3. *Loss and Advantage Calculation*: Compute the loss $L\left({\varphi }_{j}\right)$ in Eq. 2 and calculate the normalized advantage ${A}^{{\prime} }({\varphi }_{j})$ in Eq. 6.
4. *Digital Policy Update*: Perform $K$ iterations of digital optimization (Eq. 1) on the policy parameters $\theta$ using the collected $M\times B$ data points, without acquiring new measurements. The probability ratio $r\left(\varphi ;\theta \right)$ is calculated numerically in each iteration.
5. *Policy Refresh*: After $K$ updates, set ${\theta }_{{old}}=\theta$ to prepare for the next physical sampling and optimization round.
6. *Exit Condition*: Repeat the above process until the maximum number of training epochs (e.g., 50-250) is reached. Upon termination, exit the loop and output the mean of ${\pi }_{\theta }$ as the final optimized parameters for the phase mask $\varphi$.

### Implementation details

In the numerical validation of PPO-based in situ training strategy shown in Fig. 2b, we simulated a single-layer diffractive system for handwritten digit classification. Specifically, the phase-encoded object was displayed centrally using 168$\times$168 pixels with a $0-\pi$ phase range, and the trainable diffractive layer was 256$\times$256 pixels with a $0-2\pi$ phase range. The pixel size was 8 *μm*. For the in silico single-layer baseline design, used for comparison, the angular spectrum method was used to simulate propagation from the diffractive layer to the detector plane^63^, where the kernel can be written as:

7 $${H}_{z}\left({f}_{x},{f}_{y}\right)=\left\{\begin{array}{c}\exp \left(2\pi {jz}\sqrt{\frac{1}{{\lambda }^{2}}-{f}_{x}^{2}-{f}_{y}^{2}}\right),{if}\sqrt{{f}_{x}^{2}+{f}_{y}^{2}}\le \frac{1}{\lambda }\\ 0,{otherwise}\end{array}\right.$$

where $z$ is the free-space propagation distance, $\lambda$ is the wavelength, ${f}_{x}$ and ${f}_{y}$ are the spatial frequencies along x and y, respectively. For in silico simulations, we set the propagation distance $z$ as 9.6 ${cm}$ and the wavelength $\lambda =520$ ${nm}$ to match our experimental hardware, although these values were not used or necessary for our in situ reinforcement learning. The loss was calculated using a cross-entropy function on the summed intensity within 10 distinct detector areas^6^.

In the targeted energy focusing task, a diffractive layer comprising 128$\times$128 pixels, each with a size of $16\,\mu m$, was employed. The layer was operated within a $0-2\pi$ phase range. The same cross-entropy loss function used above for optical classification was used to maximize the energy concentrated within the target region while simultaneously minimizing energy in all the other 9 selected regions. The experiments also utilized a diffuser (Edmund Optics, 1° Diffusing Angle 2” x 2” Unmounted Sheet) inserted between the SLM and the image sensor, using the same optical configuration.

For the holographic image generation task, the phase pattern consisted of 256$\times$256 pixels, each with a size of $8\,{\rm{\mu }}{\rm{m}}$. The target image was 128$\times$128 pixels with a pixel size of 5.86 $\mu m$, which followed the pixel size of the camera used. In the case of aberration correction for novel image generation from random noise (Fig. 6), we initialized the process using an optical generative model^64^. Subsequently, we implemented in situ optimization of the central 512$\times$512 pixels with a pixel size of $16\,\mu m$. A 438$\times$438-pixel region of interest (ROI), with a pixel size of $5.86\,\mu m$, was selected and then resized to the target image dimension of $320\times$320 pixels (pixel size $8\,\mu m$). For both the holographic image generation and aberration correction tasks, the energy level of the measured image was first normalized to that of the target image. The Mean Squared Error (MSE) was then employed to calculate the loss between the normalized measurement and the target image. We additionally quantified the generated gratings using a contrast metric. The grating contrast $C$ was computed using:

8 $$C=\frac{{I}_{max}-{I}_{min}}{{I}_{max}+{I}_{min}}$$

To compute ${I}_{\max }$ and ${I}_{\min }$, the intensity values of the captured images were first averaged over a 10-pixel-wide stripe orthogonal to the grating direction, then the maximum and minimum values of this averaged profile were extracted across the entire image.

For each optimization round, we generated $M$ different phase masks. For the energy focusing task, we selected $M=32$. For more complex tasks like holographic image generation and aberration correction, $M=64$ was used. As for the optical image classification task, we set $M=128$. In the optical classification task, each of these phase profiles was tested with $B=8$ different input handwritten digits, resulting in $N=1024$ total physical measurements per round. For all other tasks, each mask was measured once ($B=1$). The collected data were then used to optimize the policy ${\pi }_{\theta }$ following the training steps 1-6 outlined earlier.

As discussed before, we reused the same measurements to update the training parameters multiple times—specifically, we performed $K=4$ digital optimization steps in Eq. 1 using the same batch of measured data. This strategy significantly reduced the number of new physical measurements required per update, which is particularly important in hardware-in-the-loop optimization, where each measurement can be time-consuming. In our case, capturing a single image took ~120 ${ms}$, primarily due to the response time of the SLM and the exposure needed to achieve a sufficient signal-to-noise ratio. We used a small adjustment factor ($\epsilon =0.02$) in the PPO algorithm to control how much the phase distribution is allowed to change at each step. This clipping mechanism (Eq. 1) helped stabilize training by preventing large, abrupt changes to the policy.

The optical setup consisted of a laser source (Fianium, 520 ${nm}$), different types of SLMs and cameras depending on the task. For all the tasks, we used an SLM (HOLOEYE PLUTO-2.1, pixel pitch 8 $\mu m$, resolution $1920\times 1080$) as the trainable optical component being controlled by RL. In the aberration correction task, the encoded input phase pattern was displayed on another SLM (Meadowlark XY Phase Series, pixel pitch 8$\mu m$, resolution $1920\times 1200$). A CMOS camera (PointGrey BFLY-PGE-12A2M-CS) was used for capturing the resulting light intensity patterns in the optical classification task, while another camera (Basler ace acA1920-40gm) was used for other tasks. All digital training was implemented using Python (version 3.9.21) and PyTorch (version 2.1.2, Meta Platforms Inc.). This was performed on a workstation equipped with an NVIDIA GeForce RTX 2080 Ti GPU, an AMD Ryzen 5 1600X CPU, and 32 GB of RAM. For optimizing the policy parameters, we utilized the Adam optimizer with a learning rate of 0.3.

To maintain alignment and ensure robust performance during long runs, we incorporated a periodic calibration step for training the optical classifier. This procedure corrected for minor system shifts caused by mechanical vibrations or environmental factors. We periodically projected a fixed random phase pattern and calculated the autocorrelation of the resulting image against a reference captured at the beginning. By estimating the spatial shift from this autocorrelation, we dynamically updated the camera’s region of interest, ensuring the in situ training process remained stable and efficient.

## Supplementary information

Supplementary Information

## Data Availability

All the data and methods needed to evaluate the conclusions of this work are presented in the main text. Additional data can be requested from the corresponding author (A.O.). The codes used in this work use standard libraries and scripts that are publicly available in PyTorch.
