# Supplementary material for: Model-free optical processors using in situ reinforcement learning with proximal policy optimization
Source: Light Sci Appl. 2026 Jan 1;15:32. doi: 10.1038/s41377-025-02148-7 (PMC12756285; doi:10.1038/s41377-025-02148-7)
Supplement: Supplementary file 1 — Supplementary Information [file 41377_2025_2148_MOESM1_ESM.pdf]

Supplementary Information for

Model-free Optical Processors using *In Situ* Reinforcement Learning with Proximal Policy Optimization

Yuhang Li[1,2], Shiqi Chen[1,2], Tingyu Gong[3], and Aydogan Ozcan[*,1,2,4]

[1]Electrical and Computer Engineering Department, University of California, Los Angeles, CA, 90095, USA.

[2]California NanoSystems Institute (CNSI), University of California, Los Angeles, CA, USA.

[3]Computer Science Department, University of California, Los Angeles, 90095, USA.

[4]Bioengineering Department, University of California, Los Angeles, 90095, USA.

[*]Correspondence: Aydogan Ozcan. Email: ozcan@ucla.edu

This Supplementary Information file includes:

- Supplementary Figures S1-S4

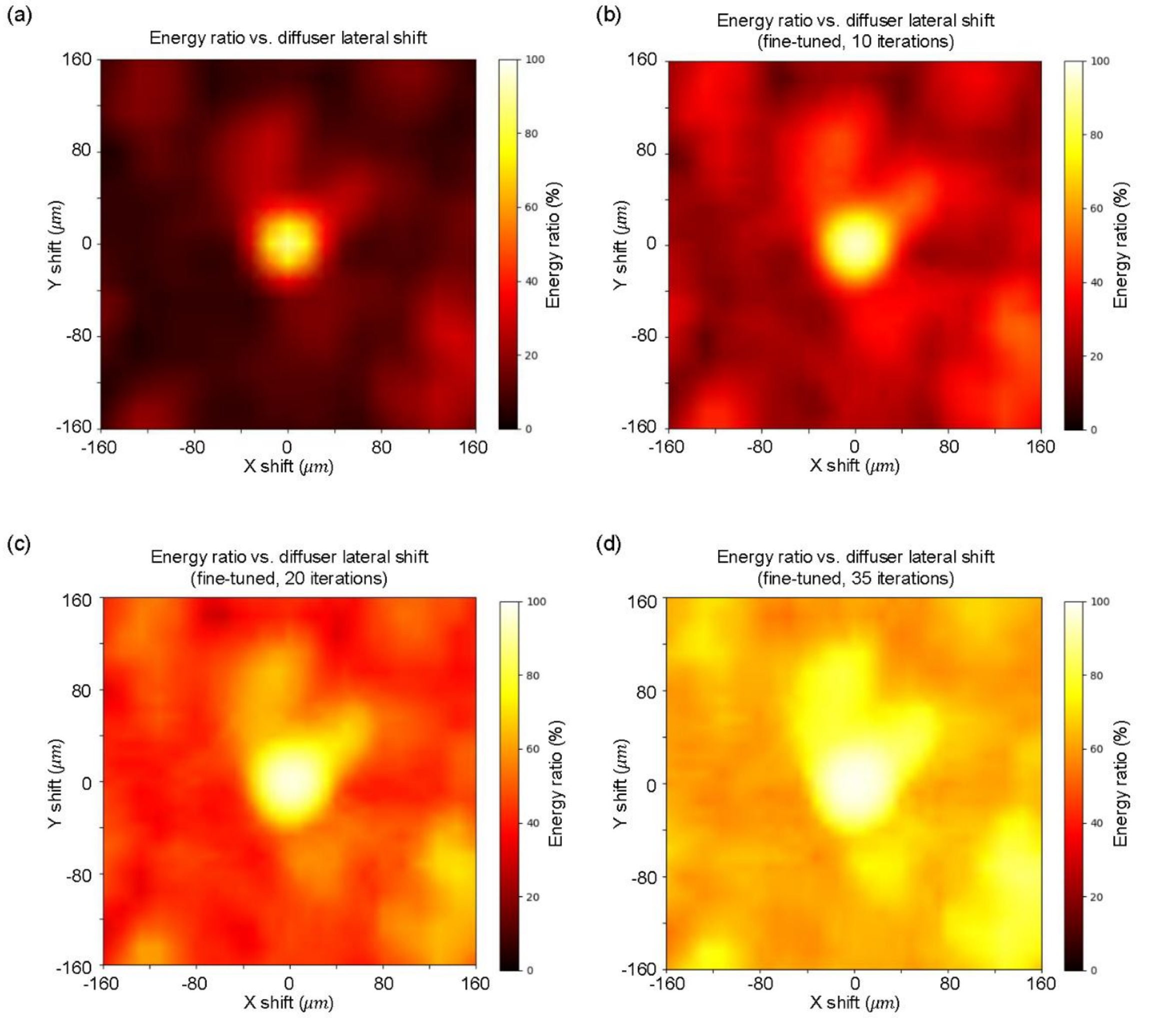

Figure S1. Simulation of reinforcement learning results for *in situ* optimization of targeted energy focusing with a random, unknown diffuser inserted between the SLM and the image sensor plane, corresponding to the setup shown in the main text, Fig. 4. (a) Energy ratio vs. diffuser lateral shift (in X and Y) using the initially trained phase pattern. (b–d) Energy ratio maps after fine-tuning with 10, 20, and 35 iterations, respectively. These simulation results demonstrate that the PPO-based method can adaptively recover high energy ratios under large amounts of lateral diffuser displacements, with more iterations leading to improved robustness across a wider range of lateral shifts.

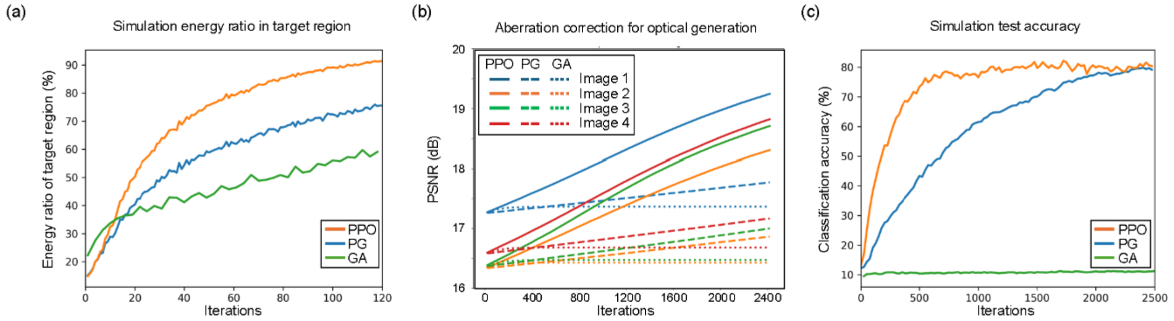

Figure S2. Numerical comparisons of PPO, PG, and GA across different optical tasks. Three methods were compared using: (a) Targeted energy focusing through a random, unknown diffuser; (b) Simulation of aberration correction for synthesizing novel images using an optical generative model. Phase aberration was numerically added to the encoded phase patterns, and it was modeled using random Zernike polynomials added with random phase noise, fixed across all the generated images. (c) Classification accuracy for an all-optical diffractive image classifier. The results show that PPO achieves faster convergence and higher final performance compared to PG, while both PPO and PG outperformed GA.

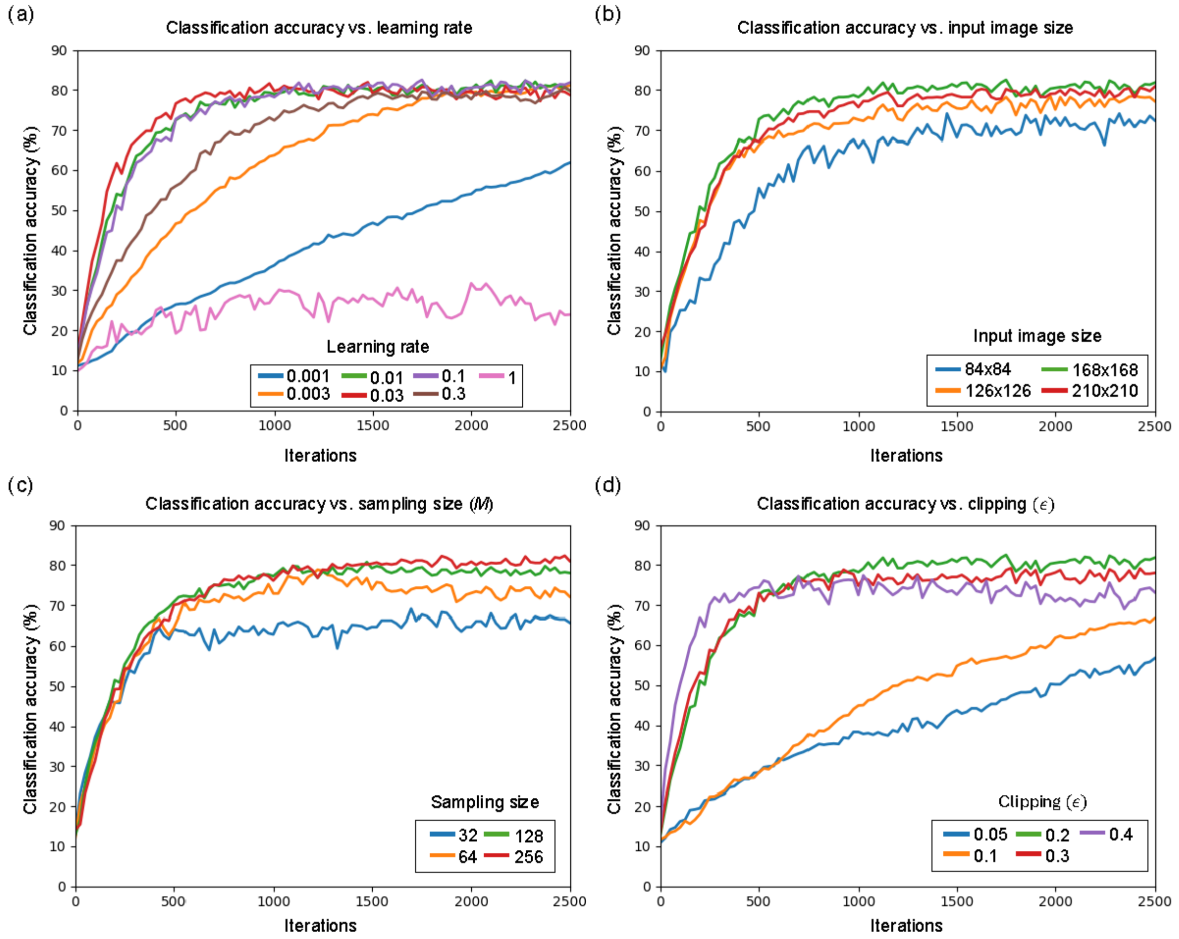

Figure S3. Simulation results showing the impact of different hyperparameters on PPO-based *in situ* training of an all-optical diffractive image classifier. Classification accuracy vs. (a) learning rate, (b) input image size, (c) sampling size (M), and (d) clipping parameter (ϵ).

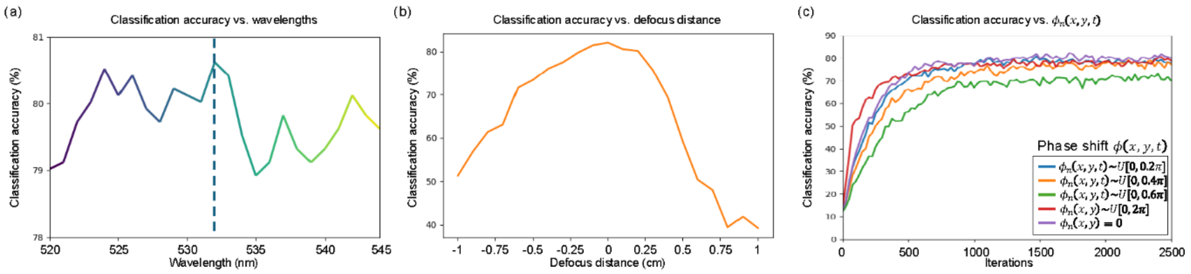

Figure S4. Robustness analysis of the PPO-trained all-optical diffractive image classifier under different conditions. Simulation classification accuracy as a function of (a) illumination wavelength changes, and (b) defocus distance. The model was trained with a single wavelength (532 nm) and zero defocus, and tested under varying wavelengths and defocus levels/distances. (c) Simulation of the classification accuracy vs. additional random phase shifts $\phi_n(x, y, t)$ applied to the diffractive layer, where these phase perturbations $\phi_n(x, y, t)$ were included during both training and testing, with the presented results showing test accuracy across different learning iterations.
